# Supplementary material for: A Low Iron Diet Protects from Steatohepatitis in a Mouse Model
Source: Nutrients. 2019 Sep 10;11(9):2172. doi: 10.3390/nu11092172 (PMC6769937; doi:10.3390/nu11092172)
Supplement: Supplementary file 1 [file nutrients-11-02172-s001.pdf]

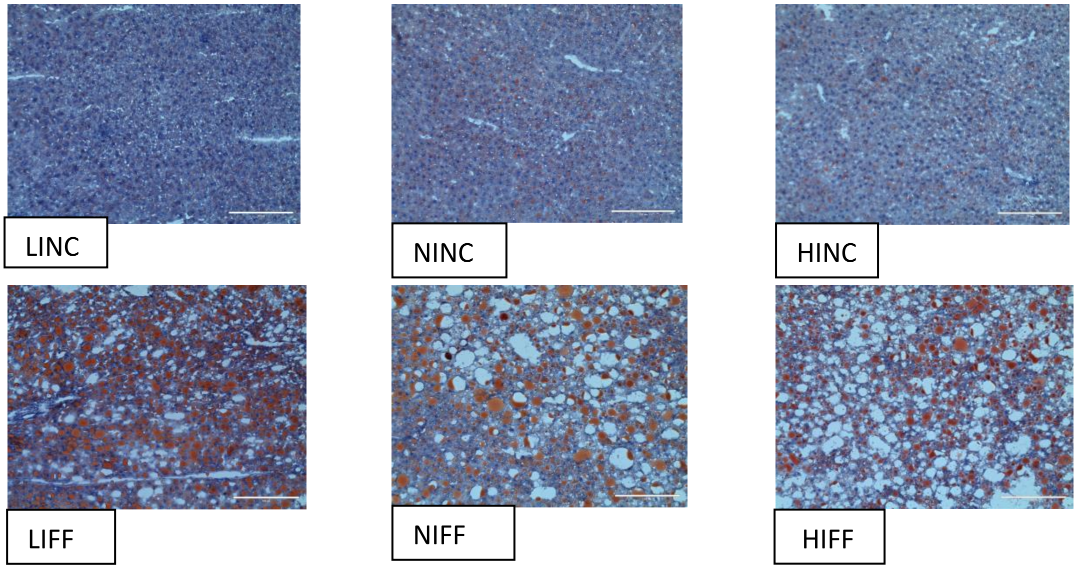

Supplementary Figure 1: Oil Red O stain on paraffin-embedded liver histology show that lipid droplets (red color) are larger in the fast food diet group (bottom panel) after 5-6 months on diet.

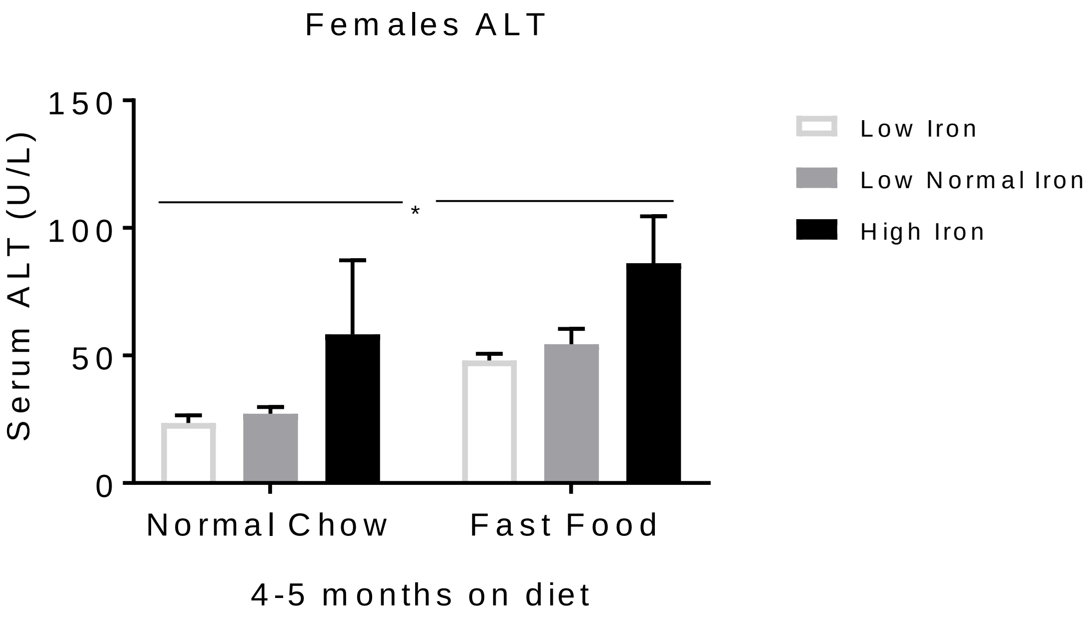

Supplementary Figure 2: Female C57Bl6 mice showed the same trend of increasing serum ALT with iron, although the overall ALT values were lower than that seen in the males. The FF group had significantly (p=0.018) higher ALT levels, suggesting that both iron and fat worsen liver injury in mice, in both males and females. n=5 mice per group. Shown are means ± SE. p values were calculated with ordinary two-way ANOVA with post hoc Tukey's multiple comparisons test.* 0.01≤p≤0.05.

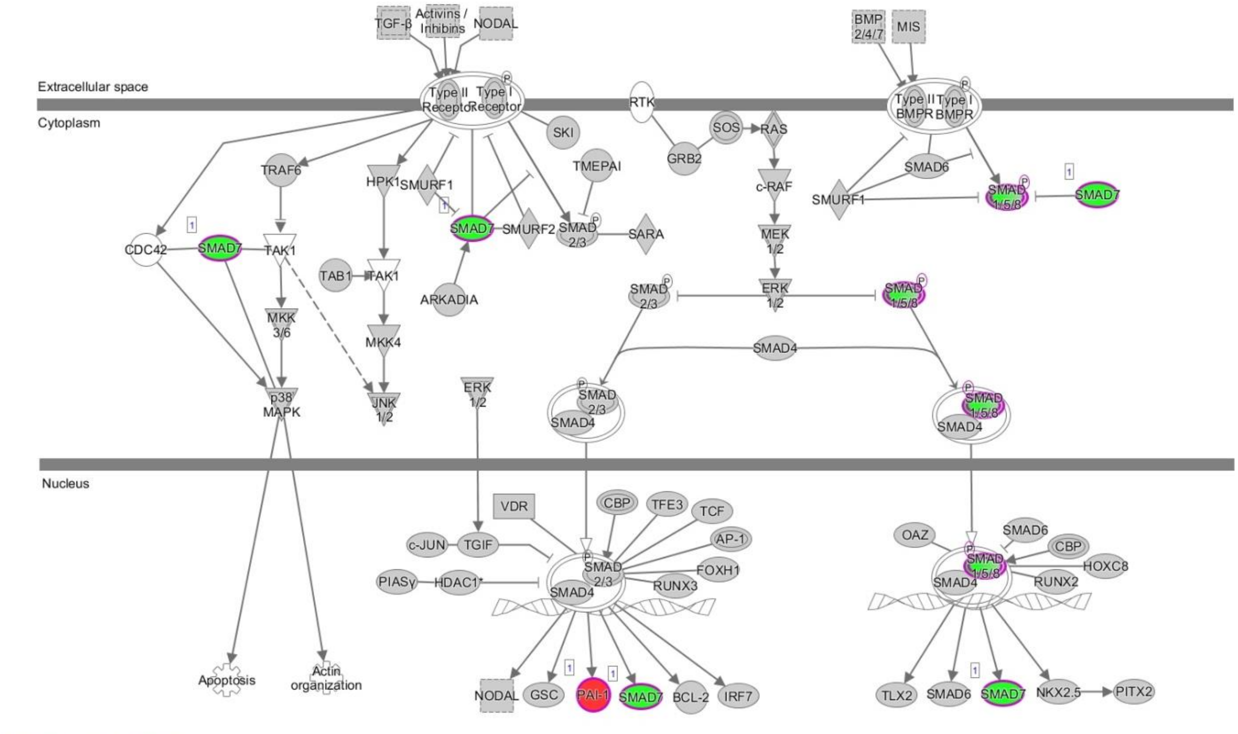

© 2000-2018 QIAGEN. All rights reserved.

Supplementary Figure 3: Hepatic TGF-β signaling is regulated by iron as indicated by our RNA-seq data. Green color means downregulation; red color means upregulation of genes in our dataset. LIFF versus HIFF liver transcriptomes at 5-6 months on diet were used for the analysis; functional annotation and pathway enrichment was done with DAVIDv6.8 and Ingenuity Pathway Analysis (IPA) pathway visualization tool. Grey are dataset molecules that do not meet the analysis criteria, and white are those not present in our differentially expressed genes (DEG) dataset. SMAD7, an inhibitor of hepcidin expression and negative regulator of TGF-β signaling pathway, is downregulated in high iron FF group; while PA-1 or serpine, known to contribute to tissue fibrosis, is elevated as compared to low iron FF group.

a.

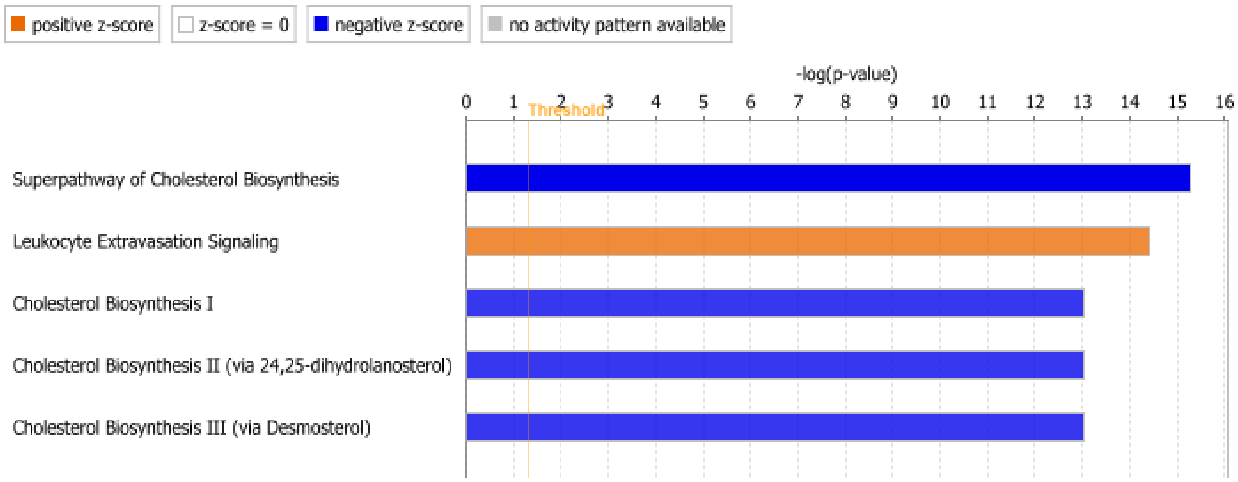

b.

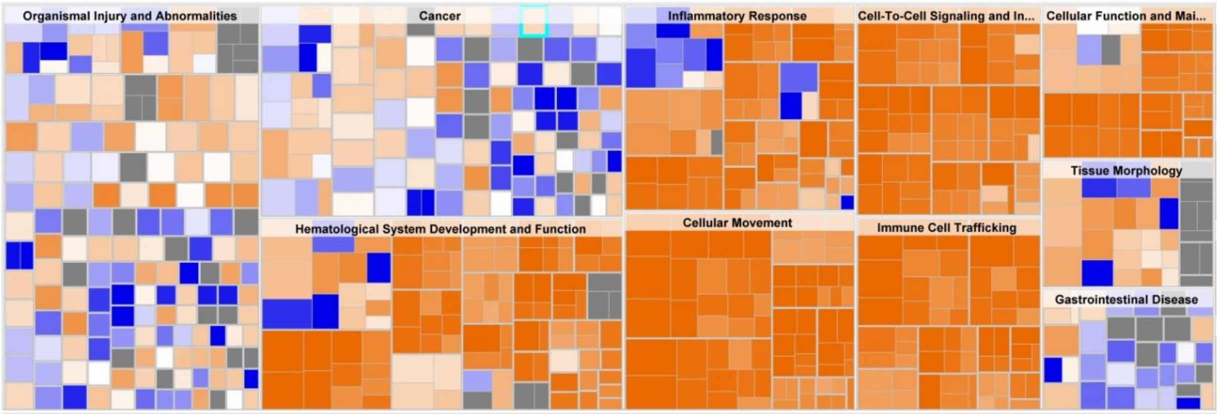

Supplementary Figure 4: Effect of FF diet on hepatic transcriptome by comparing liver transcriptomes of mice on normal iron (NI) and normal iron FF (NIFF) diets for 5-6 months and pathway enrichment analysis done using Ingenuity Pathway Analysis (IPA) core analysis. (A) The top 5 canonical pathways altered by the FF diet. Blue indicates negative z-score i.e. predicted inhibition of the pathways; and orange indicates positive z-score i.e. predicted activation of the pathway in FF diet. Length of the bar corresponds to p value of the prediction. Thus, cholesterol biosynthetic pathways are the most potently inhibited in FF diet, while inflammation-related leukocyte extravasation signaling is activated when compared to normal chow. (B) Organismal injury and abnormalities and cancer are the largest high-level functional

categories enriched in our dataset of NI vs NIFF at 5-6 months on diet. Inflammatory response, cellular movement, cell-to-cell signaling and immune cell trafficking are predicted to be trending towards an increase in the FF group. Intensity of the colors indicates prediction strength. The size of the squares reflects associated log of the calculated p-value.

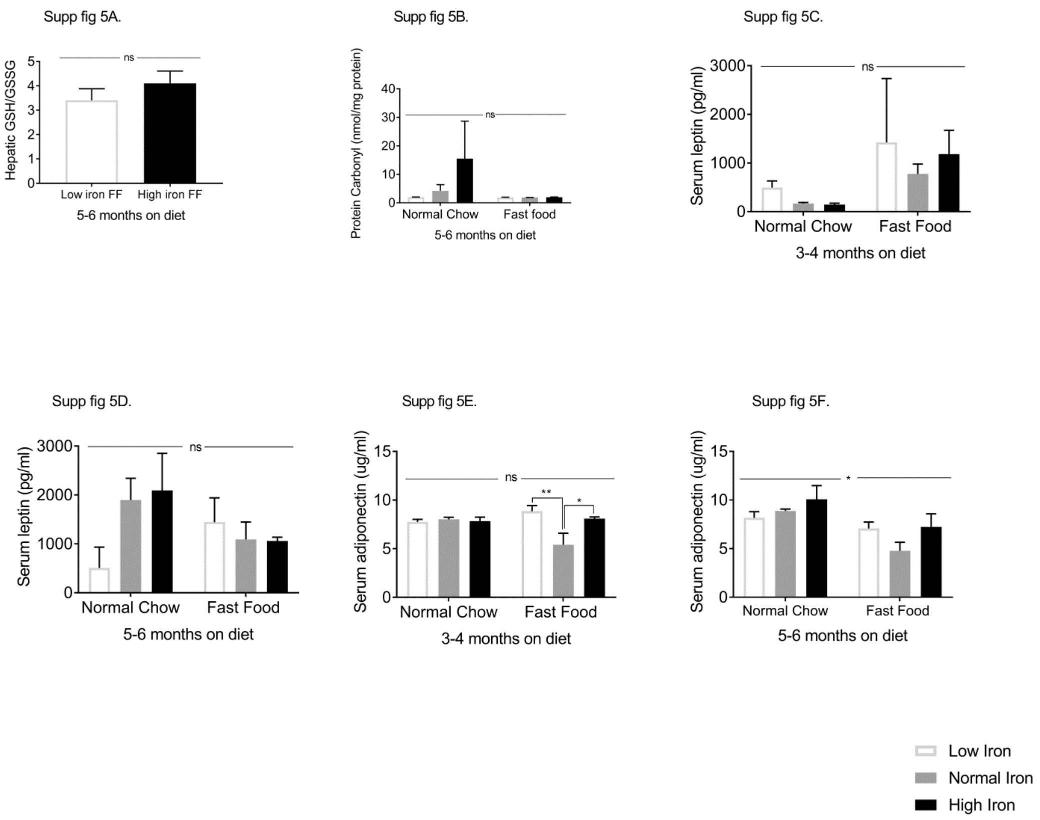

Supplementary Figure 5: Oxidative stress was measured by (A) quantifying reduced glutathione (GSH) and GSSG using liquid chromatography-mass spectrometry (LC-MS), in livers of mice on FF diet for 5-6 months. Redox ratio= (GSHt- 2*GSSG)/ GSSG]. Statistical significance was calculated using unpaired two-tailed t-test with Welch's correction. ns= not significant, $p \geq 0.05$ (n=4 mice per group).; and (B) Protein carbonyl content in the liver tissue was measured using commercial assay kits. Statistical test used was ordinary two-way ANOVA with post hoc Tukey's multiple comparisons test. ns= not significant. (n=3 mice per group). (C-D) Serum adipokines, leptin (C,D) and adiponectin (E,F), were quantified using commercial assay kits. There is no significant effect of iron, although a dosage-dependent trend is observed. p values were calculated with ordinary two-way ANOVA with post hoc Tukey's multiple comparisons test. * $0.01 \leq p \leq 0.05$, ns=not significant (n= 2-3 mice per group). Shown are means ± SE.

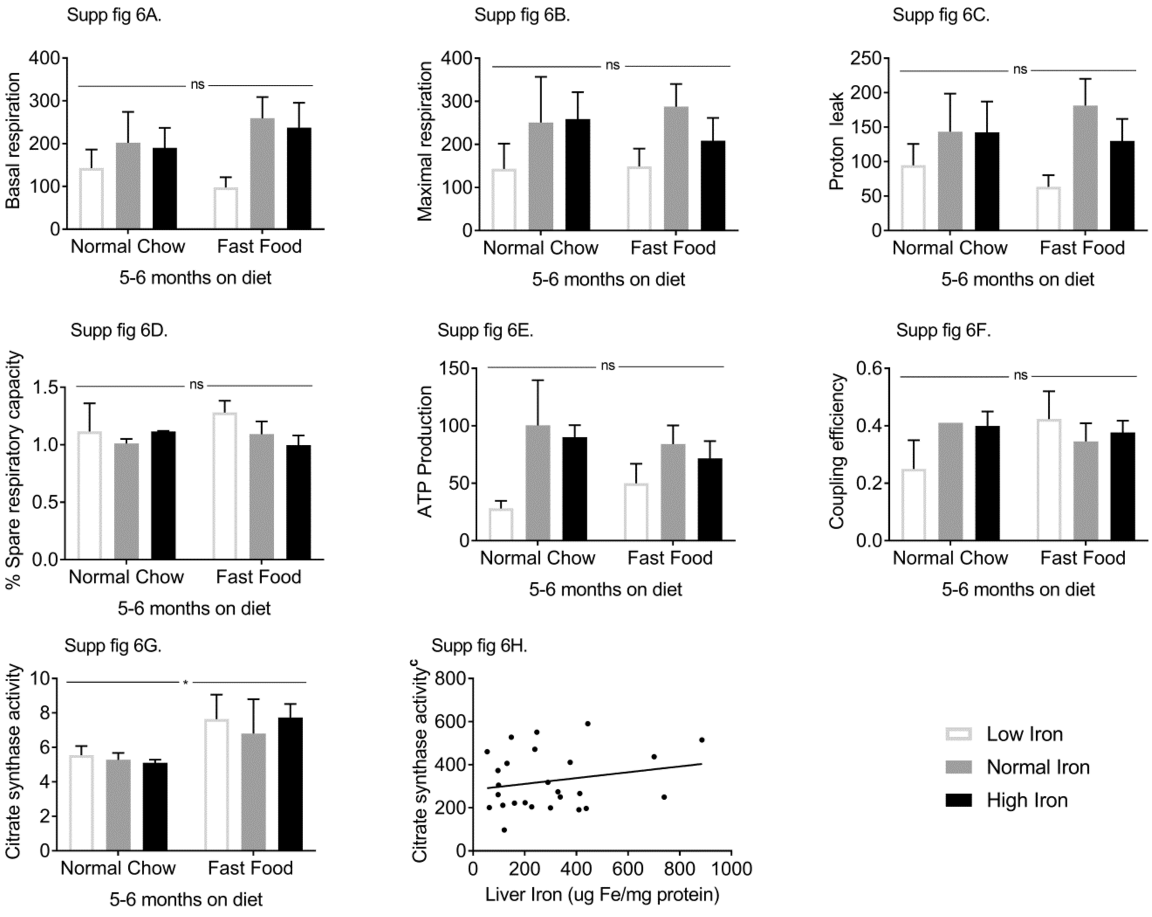

Supplementary Figure 6: Indices of mitochondrial function (A-F) Key parameters of metabolic function in freshly isolated liver mitochondria were studied using Seahorse technology (n=2-5 per group). (G) Citrate synthase activity, as a surrogate measure of mitochondrial mass, indicates no loss of intact hepatic mitochondrial mass. p values were calculated with ordinary two-way ANOVA with post hoc Tukey's multiple comparisons test. ns or not significant i.e. $p \geq 0.05$, * $0.01 \leq p \leq 0.05$. Shown are means ± SE. (H) In clinical liver biopsies from patients undergoing bariatric surgery, citrate synthase activity was not significantly correlated to hepatic iron. Linear regression model, $r^2=0.04684$, $p=0.2883$. Each dot indicates a single patient.

Supp fig 7A.

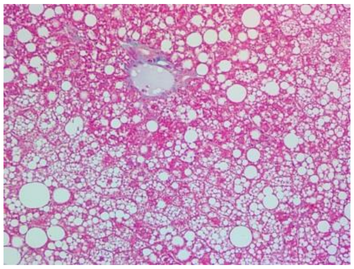

Supp fig 7B.

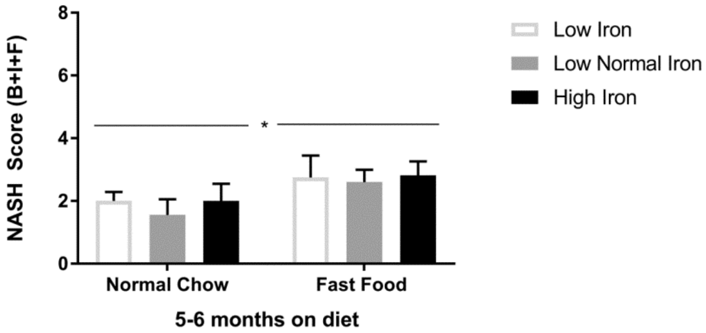

Supp fig 7C.

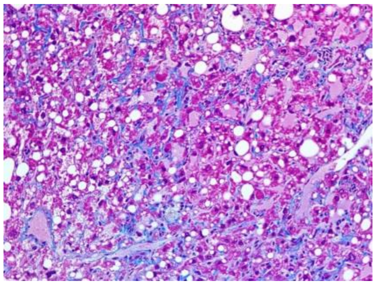

Supplementary Figure 7: Liver histology (A) Trichrome staining at 5-6 months on diet does not show collagen deposition (blue color) for tissue fibrosis. (B) Qualitative NASH scoring was calculated as a sum of individual scores of three parameters -hepatocyte ballooning or B; inflammation or I; and fibrosis or F; on a 0-3 scale for each parameter. B and I were scored on H&E stained slides while F was scored on Masson's trichrome stain. Total NASH score range is

0-8. Detailed scoring criteria are as described in Supplementary table 6. p values were calculated with ordinary two-way ANOVA with post hoc Tukey's multiple comparisons test. * $0.01 \leq p \leq 0.05$. Shown are means ± SE. (C) Trichrome staining at 14 months on diet shows significant collagen deposition (blue color) indicating severe tissue fibrosis.

Supplementary Table 1: Top KEGG pathways enriched in the DEGs as annotated by Database for Annotation, Visualization and Integrated Discovery (DAVID) v6.8

Category	Term	Count	PValue	Benjamini	FDR
**LIFF vs HIFF at 3-4 months on diet**					
KEGG_PATHWAY	mmu04350:TGF-β signaling pathway	7	5.69E-06	6.09E-04	0.0064
KEGG_PATHWAY	mmu04550:Signaling pathways regulating pluripotency of stem cells	6	8.49E-04	0.0443	0.9496
**LIFF vs HIFF at 5-6 months on diet**					
KEGG_PATHWAY	mmu04350:TGF-β signaling pathway	5	2.25E-05	0.0014	0.0228

Supplementary Table 2: Gene Ontology (GO) pathways enriched in the DEGs as annotated by Database for Annotation, Visualization and Integrated Discovery (DAVID) v6.8

Category	Term	Count	PValue	Benjamini	FDR
**LIFF vs HIFF at 3-4 months on diet**					
**GOTERM_CC_DIRECT**	GO:0005615~extracellular space	17	2.26E-05	0.0024	0.0254
**GOTERM_CC_DIRECT**	GO:0034361~very-low-density lipoprotein particle	4	3.22E-05	0.0017	0.0362
**GOTERM_CC_DIRECT**	GO:0005576~extracellular region	17	1.42E-04	0.0051	0.1599
**GOTERM_CC_DIRECT**	GO:0042627~chylomicron	3	8.20E-04	0.0219	0.9193
**LIFF vs HIFF at 5-6 months on diet**					
**GOTERM_CC_DIRECT**	GO:0005576~extracellular region	8	0.0039	0.2322	3.9643
**GOTERM_CC_DIRECT**	GO:0005615~extracellular space	7	0.0080	0.2371	7.9536

Supplementary Table 3: Top upstream regulators upon analysis of DEG dataset between LIFF and HIFF using Ingenuity Pathway Core Analysis

Upstream regulator	Name	Type of molecule	Predicted activation state	Activation z-score
**LIFF vs HIFF at 3-4 months on diet (Activation z-score >3, -3<)**				
Il1b	interleukin 1 beta	cytokine	activated	4.54
Tnf	tumor necrosis factor	cytokine	activated	3.64
Ifng	interferon gamma	cytokine	activated	3.37
Il1a	interleukin 1 alpha	cytokine	activated	3.27
Il6	interleukin 6	cytokine	activated	3.14
Csf1	colony stimulating factor 1	cytokine	activated	3.08
Pdgfbb	platelet derived growth factor subunit A&B complex	complex	activated	3.06
Il1rn	interleukin 1 receptor antagonist	cytokine	inhibited	-3.10
**LIFF vs HIFF at 5-6 months on diet (Activation z-score >2, -2<)**				
Gdf2	growth differentiation factor 2	growth factor	activated	2.40
Bmp15	bone morphogenetic protein 15	growth factor	activated	2.24
Gdf9	growth differentiation factor 9	growth factor	activated	2.24
Acvrl1	activin A receptor like type 1	kinase	activated	2.17
Bmp2	bone morphogenetic protein 2	growth factor	activated	2.08
Fst	follistatin	other	inhibited	-2.00
Pdx1	pancreatic and duodenal homeobox 1	transcriptional regulator	inhibited	-2.00

Supplementary Table 4: Top Toxicological functions based on p-value generated by IPA

Categories	Diseases or Functions Annotation	p-value
**LIFF vs HIFF at 3-4 months on diet**		
Hepatocellular carcinoma, Liver hyperplasia/hyperproliferation	Hepatocellular carcinoma	9.57E-11
Liver inflammation/Hepatitis	Inflammation of liver	2.05E-08
Liver Steatosis	Hepatic steatosis	4.29E-07
Increased levels of alkaline phosphatases	Increased activation of alkaline phosphatases	9.93E-06
Liver Fibrosis	Fibrosis of liver	1.44E-05
Liver Damage	Liver damage	1.78E-05
**LIFF vs HIFF at 5-6 months on diet**		
Increased Levels of Red Blood Cells	Increased Levels of Red Blood Cells	7.58E-04
Liver Proliferation	Proliferation of hepatocytes	3.87E-03
Liver Steatosis	Hepatic steatosis	5.33E-03
Increased levels of alkaline phosphatases	Increased activation of alkaline phosphatases	6.64E-03
Liver Inflammation/ Hepatitis	Inflammation of liver	7.76E-03
Liver Dysplasia	Liver dysplasia	1.03E-02

Supplementary Table 5: The 10 common elements that are affected by both, iron and FF diet at 5-6 months on diet

ENSEMBL Gene ID	Gene Symbol	Gene Name
ENSMUSG00000061780	Cfd	complement factor D (adipsin)
ENSMUSG00000033854	Kcnk10	potassium channel, subfamily K, member 10
ENSMUSG00000092008	Cyp2c69	cytochrome P450, family 2, subfamily c, polypeptide 69
ENSMUSG00000039809	Gabbr2	gamma-aminobutyric acid (GABA) B receptor, 2
ENSMUSG00000098973	Mir6236	microRNA 6236
ENSMUSG00000057969	Sema3b	sema domain, immunoglobulin domain (Ig), short basic domain, secreted, (semaphorin) 3B
ENSMUSG00000032357	Tinag	tubulointerstitial nephritis antigen
ENSMUSG00000022797	Tfrc	transferrin receptor
ENSMUSG00000037411	Serpine1	serine (or cysteine) peptidase inhibitor, clade E, member 1
ENSMUSG00000025004	Cyp2c40	cytochrome P450, family 2, subfamily c, polypeptide 40

Supplementary Table 6: Criteria for NASH scoring on liver histology based on the publication by Kleiner et al, 2005 (100).

Item	Score	Extent	Definition and Comment
Steatosis	0	<5%	Refers to amount of surface area involved by steatosis as evaluated on low to medium power examination; minimal steatosis (<5%) receives a score of 0 to avoid giving excess weight to biopsies with very little fatty change.
	1	5-33%	
	2	>33-66%	
	3	>66%	
Lobular Inflammation	0	No foci	Acidophil bodies are not included in this assessment, nor is portal inflammation.
	1	<2 foci/200x	
	2	2-4 foci/200x	
	3	>4 foci/200x	
Hepatocyte Ballooning	0	None	
	1	Few balloon cells	The term "few" means rare but definite ballooned hepatocytes as well as cases that are diagnostically borderline
	2	Many cells/prominent ballooning	Most cases with prominent ballooning also had Mallory's hyalin, but Mallory's hyaline is not scored separately for the NAS

Fibrosis Stage (Evaluated separately from NAS)

Item	Score	Extent	Definition and Comment
Fibrosis	0	None	

1	Perisinusoidal or periportal	
1A	Mild, zone 3, perisinusoidal	"delicate" fibrosis
1B	Moderate, zone 3, perisinusoidal	"dense" fibrosis
1C	Portal/periportal	This category is included to accommodate cases with portal and/or peri portal fibrosis without accompanying pericellular/perisinusoidal fibrosis
2	Perisinusoidal and portal/periportal	
3	Bridging fibrosis	
4	Cirrhosis	

Note: Steatosis parameter is not included in the graph (Supplementary Figure 6).
